# Development of novel predictive miRNA/target gene pathways for colorectal cancer distance metastasis to the liver using a bioinformatic approach

**DOI:** 10.1371/journal.pone.0211968

**Published:** 2019-02-26

**Authors:** Precious Takondwa Makondi, Po-Li Wei, Chien-Yu Huang, Yu-Jia Chang

**Affiliations:** 1 International PhD Program in Medicine, Taipei Medical University, Taipei, Taiwan, ROC; 2 Graduate Institute of Clinical Medicine, College of Medicine, Taipei Medical University, Taipei, Taiwan, ROC; 3 Division of Colorectal Surgery, Department of Surgery, Taipei Medical University Hospital, Taipei Medical University, Taipei, Taiwan; 4 Department of Surgery, College of Medicine, Taipei Medical University, Taipei, Taiwan; 5 Cancer Research Center and Translational Laboratory, Department of Medical Research, Taipei Medical University Hospital, Taipei Medical University, Taipei, Taiwan; 6 Graduate Institute of Cancer Biology and Drug Discovery, Taipei Medical University, Taipei, Taiwan; 7 Division of General Surgery, Department of Surgery, Shuang Ho Hospital, Taipei Medical University, Taipei, Taiwan, ROC; University of South Alabama Mitchell Cancer Institute, UNITED STATES

## Abstract

**Background:**

Liver metastases are the major cause of colorectal cancer (CRC)-related deaths. However, there is no reliable clinical predictor for CRC progression to liver metastasis. In this study, we investigated possible predictors (miRNAs and biomarkers) for clinical application.

**Methodology:**

The Gene Expression Omnibus (GEO) datasets GSE49355, GSE41258 and GSE81558 for genes and GSE54088 and GSE56350 for miRNAs were used to identify common differentially expressed genes (DEGs) and miRNAs between primary CRC tissues and liver metastases. The identified miRNAs and their targets from the DEGs were verified in datasets comprising gene, miRNA and miRNA exosome profiles of CRC patients with no distant metastases (M0) and distant metastases (M1); the interaction networks and pathways were also mapped.

**Results:**

There were 49 upregulated and 13 downregulated DEGs and 16 downregulated and 14 upregulated miRNAs; between the DEGs and miRNA targets, there were five upregulated and four downregulated genes. MiR-20a was strongly correlated with the status of liver metastasis. MiR-20a, miR499a, and miR-576-5p were highly correlated with the metastatic outcomes. MiR-20a was significantly highly expressed in the M1 group. In an analysis of the miRNA target genes, we found that CDH2, KNG1, and MMP2 were correlated with CRC metastasis. We demonstrated a new possible pathway for CRC metastasis: miR-576-5p/F9, miR20a/MMP2, CTSK, MMP3, and miR449a/P2RY14. The regulation of IGF transport and uptake by IGFBPs, extracellular matrix organization, signal transduction and the immune system were the enriched pathways.

**Conclusion:**

This model can predict CRC to liver metastases and the pathways involved, which can be clinically applicable.

## Introduction

Colorectal cancer (CRC) is the third most frequently diagnosed cancer worldwide and ranks fourth as a cause of all cancer-related deaths[1–4]. The major problem in the management of CRC is that approximately 23% of patients already have metastatic disease at presentation, and approximately 40% of CRC patients develop metastases during the course of treatment, with the liver being the most common metastatic site (over 50% of CRC metastases)[5–7]. These characteristics lead to the death of more than half of CRC patients and contribute to a low 5-year overall survival rate of less than 10% in those with metastatic colorectal cancer (mCRC); however, in the 15% of patients who benefit when liver metastases are diagnosed and treated early, the 5-year overall survival rate can be 40–74%[6–12]. Therefore, the detection of early liver metastasis is crucial to improving patient survival, but it is limited by the current TNM classification because of its poor predictive value for metastasis; this method is not sufficient for determining the likelihood of survival[13, 14]. Recently, many studies have focused on identifying biomarkers that can predict the metastatic site of the primary tumor[15, 16].

MicroRNAs (miRNAs) are small non-protein coding single-stranded RNAs (between 17 and 25 nucleotides)[17] that regulate the expression of different gene targets at the post-transcriptional level, suppressing mRNAs and causing mRNA degradation[17, 18]. More than one-third of human genes and pathways are regulated by miRNAs, and their alterations are associated with different cancers[18, 19]. Therefore, miRNAs can function as oncogenes or tumor suppressors due to their effects on target genes[20]. MiRNA profiling or signatures of cancer have been shown to identify potential tumor subtypes, diagnose cancer, determine treatment plans and predict patient outcome[21–23]. The role of miRNAs in metastatic development has also been demonstrated in different cancers, including CRC[24]. These molecules have been associated with metastasis of CRC to the liver[15, 25], and they have been shown to be highly tumor and tissue specific[26, 27]. As most of the studies involving miRNAs and the metastatic pathway in CRC used *in vitro* and mouse models[24], with only a few relying on tissue-based experiments[28], the mechanisms and the roles of miRNAs in liver metastasis of CRC are not fully understood. Therefore, there is a need to comprehensively explore the roles of miRNAs in the development of liver metastasis from primary CRC tumors.

Only a few studies have compared miRNAs and their target gene changes between primary colon tumors and metastatic tissues to identify the miRNAs and target genes implicated in cancer progression[29–31]. Thus, studies assessing miRNA and gene expression levels between primary and liver metastases were searched in the Gene Expression Omnibus (GEO) to identify the miRNAs and gene targets implicated in metastatic colonization of the liver in CRC. The identified miRNAs and gene targets were validated using datasets with clinical information about patient progression and survival. The findings of this study identified a miRNA and a gene signature that can predict liver metastases in CRC patients.

## Materials and methods

### Microarray gene and miRNA expression datasets

The GEO (www.ncbi.nlm.nih.gov/geo/)[32], a public data repository for high-throughput gene and miRNA expression datasets, was used to search for datasets that had gene and miRNA expression profiles between primary colon tumors (CTs) and hepatic metastases (HM). Three gene microarrays and two miRNA datasets were identified and downloaded. The gene microarray data sets included GSE49355, which had 57 samples which included normal colon (18), CT (20) and HM (19). From this, 13 pairs of samples between CTs and HM were selected and included, and all the normal, and CT and HM samples which were not in pairs were excluded. The expression profiling for the 26 samples was conducted on platform GPL96 [HG-U133A] Affymetrix Human Genome U133A Array chip, which contained 22200 probes corresponding to approximately 12700 genes[16]. The second dataset was GSE41258, which had 390 expression arrays comprising normal colon(54), polyps (49), CT (186), microadenomas (2), normal liver (13), HM (47), normal lung (7), lung metastases (20) and colon cancer cell lines (12) [33], where 39 paired CT and HM tissues were found, selected and included; and all other non-paired CT and HM samples were excluded together with other expressions. The expression profiling was conducted on Affymetrix human U133A chips, platform GPL96, which contained over 1,000,000 unique oligonucleotide features covering more than 39,000 transcript variants, representing greater than 33,000 of the best characterized human genes. The other dataset included was GSE81558, which had 51 tissue specimens including 9 from normal mucosa, 23 sporadic colorectal adenocarcinomas and 19 liver metastases obtained from 23 patients with metastatic lesions[34], therefore 19 paired CT and HM were included.It was generated from platform GPL15207, [PrimeView] Affymetrix Human Gene Expression Array. The miRNA datasets were GSE54088, which had 34 expression profiles from normal colon (10), CT (9), normal liver (8), HM (6) and other metastases (1) where paired 5 CTs and 5 HM, we found and included for analysis[30, 31], sequenced on platform GPL8178, Illumina Human v1 MicroRNA expression beadchip panel containing 735 microRNAs and GSE56350[15],profiled on GPL16744, OSU-CCC Human and Mouse MicroRNA Microarray Version 4.0 [condensed human miRNA version] and included 785 miRNAs.The dataset included 104 samples from 46 CT, 15 HM and 43 lymphnode metastases and only 15 paired CTs and HM samples were found and included.

### Common genes and miRNAs and the identification of common miRNA targets

To identify common differentially expressed genes (DEGs) in the three microarray datasets, we used GEO2R (www.ncbi.nlm.nih.gov/geo/geo2r)[32], an interactive web tool that can compare two or more groups of samples under the same experimental conditions in a GEO dataset, to identify DEGs or differentially expressed miRNAs (DEMs) between primary colorectal tumor and liver metastasis samples. The genes that satisfied the inclusion criteria of p<0.05 and a false discovery rate (FDR) of <0.05 were identified as DEGs and included in the study, the miRNAs that satisfied the inclusion criteria of p<0.05 and a false discovery rate (FDR) of <0.1 were identified as differentially expressed (DE) miRNAs and included in the study. Venny 2.1.0, an online web tool, was used to identify common DEGs in these three datasets and to plot Venn diagrams[35]. This tool can summarize the overlap between two to four lists to enable the observation of similarities and differences in the datasets. Each circle represents a dataset, and the overlap between the circles corresponds to the overlap between the datasets. The miRNAs were also analyzed by GEO2R between CTs and HM in the two datasets, and the Venny tool was used to identify common upregulated and downregulated DE miRNAs. The common DE miRNAs were then entered into the miRDB online software (http://mirdb.org/miRDB/)[36] to predict the miRNA targets. These miRNA targets were compared to the previously identified DEGs to identify the miRNA targets that were enriched in the gene microarray datasets. The identified miRNAs and their targets were also verified using mirDIP 4.1, and integrative database of human microRNA target predictions, which uses over 30 miRNA predicting tools including target scan, DIANA tool, miRanda and miRDP to produce an integrative score, which was statistically inferred from the obtained predictions, and was assigned to each unique microRNA-target interaction to provide a unified measure of confidence[37].

### Clinical validation of the common miRNAs and their target genes

CRC metastasizes to the liver in 60–80% of patients, and the liver is the sole organ with metastases in approximately one-third of CRC patients with distance metastases [38–40]. In addition, about 50% and 20% of patients with stage III and stage II CRC respectively, will develop liver metastases[38, 41]. This is mainly because the colon’s venous drainage is through the portal vein, which goes directly to the liver[7]. The European Colorectal Metastases Treatment Group (ECMTG) recommended a staging system of using M from the TNM classification to classify CRC into M0 for patients with no known distance metastasis and M1 for patients with known distance metastases (both liver and metastases outside of the liver)[42]. Because most follow-up clinical data do not specifically include details for specific metastatic sites, the identified common DE miRNAs from the two datasets and their targets which were shared with the DEGs identified from the three datasets were verified on their role of differentiating between CRC patients with no distant metastases (TNM stage I, II and II = M0) and those with distant metastases (TNM stage IV = M1), we used the GEO database to identify datasets that had gene and miRNA expression of patients with CRC and contained clinical and prognostic information. Dataset GSE29623 generated by Chen Et al., which contained miRNA expression from 65 cases with colon cancer with their miRNA expression signature and could differentiate stage I and stage IV primary colon cancers, was selected. In this dataset, 47 cases were classified as stage M0, while 18 were M1. For validation of the miRNA targets, the GSE39582 dataset by Malisa et al.[43], which had gene expression profiles from primary tumor samples of 750 patients with stage I to IV CRC who underwent surgery between 1987 and 2007 in seven centers, was selected. For validation, 521 patients at various stages (Stage I, II, III = M0) and 61 stage IV patients (Stage IV = M1) were included. The series matrix files for the above datasets were downloaded, and the expression profiles of the miRNAs and the target genes of interest were extracted. Box plots were plotted to show the expression status between the M0 and M1 groups, and a Mann-Whitney test was performed to calculate the p value, with p<0.05 defined as significant. To further evaluate the explanatory power of the miRNAs and their target genes, we performed logistic regression and tested the significance. The regression model was estimated based on the miRNAs and the target genes, which showed significant differences in expression between M1 and M0 group genes, and the score was calculated. This model was followed by receiver operating characteristic (ROC) analysis(36), and the area under the ROC curve (AUC), sensitivity and specificity were calculated by Youden’s method.

### Clinical validation of the common miRNAs in serum exosomes from CRC patients

With the increase in interest in circulating miRNAs as new cancer biomarkers, exosomes, which are small membrane vesicles that are secreted from various cells with their components (lipids, mRNAs, miRNAs and proteins), reflecting the original cells secreting them[44–47], have been assessed as biomarkers. The identification of exosomal miRNAs from cancer cells was suggested to provide useful biomarkers of CRC liver metastases. Therefore, the identified miRNAs were also validated in the GSE39833 dataset comprising profiled exosomal miRNA expression taken from the sera of colon cancer patients (n = 88) at various TNM stages (I; n = 20, stage II; n = 20, stage IIIa; n = 20, stage IIIb: n = 16, stage IV; n = 12) (age; 35–65 years). Briefly[48], serum exosomes were prepared using a stepwise ultracentrifugation method, and the exosome fraction was mixed with TRIzol-LS reagent (Invitrogen). The aqueous phase was collected by addition of chloroform. After addition of ethanol to the aqueous phase, it was placed in a RNeasy mini spin column (Qiagen) for the purification of total RNAs, and the total RNAs were analyzed by an Agilent Human miRNA V3 Microarray (G4470C) following the manufacturer's instructions.

### Gene-to-gene interaction and functional and pathway enrichment

To identify biological functions of miRNA targets, we used functional network analysis, and GeneMANIA (http://www.genemania.org/)[49], an online tool that was developed to predict gene or protein function based on a query of a list of proteins that share a function of interest and uses a network to predict interactions and function of genes, was applied. The networks in GeneMANIA are customizable and highlight specific functions. GeneMANIA uses references from different databases, including GEO, BioGRID, EMBL-EBI, Pfam, Ensembl, Mouse Genome Informatics, the National Center for Biotechnology Information, InParanoid, and Pathway Commons. For reactome pathway analysis, the Reactome FI Cytoscape plugin for Cytoscape 3.6.1 was used[50, 51], and pathways with FDR<0.05, p-value<0.05 and genes≥4 were selected.

## Results

### Potential miRNA-mRNA interactors in primary CRC tumors and liver metastases

**Fig 1:**
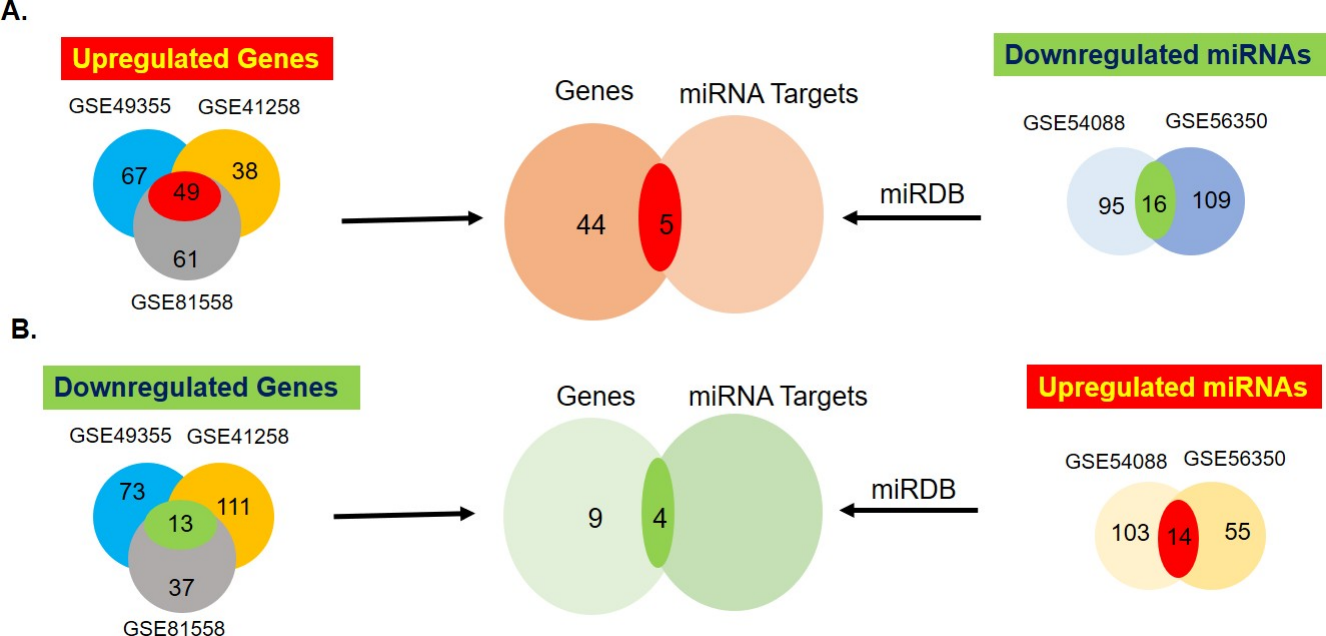
Representative diagrams of identification of microRNAs (miRNAs) and their targets between primary colorectal tissues and their hepatic (liver) metastases. The Venn diagrams demonstrate the number of common differentially expressed genes (DEGs), miRNAs and the miRNA targets (red = upregulated and green = downregulated) from different Gene Expression Omnibus (GEO) datasets.

The DEG and miRNA expression levels were simultaneously analyzed to identify potential miRNA-mRNA interactors between CTs and HM by GEO2R. There were 62 common DEGs, of which 49 were upregulated and 13 were downregulated, in the 3 mRNA datasets (Fig 1, S1–S4 Tables). In the two miRNA datasets, there were a total of 30 common miRNAs, with 16 upregulated and 14 downregulated (Fig 1 and Table 1). To identify miRNA targets among the identified DEGs, we used a miRDB predictor, and the targets of upregulated miRNAs were compared with downregulated DEGs, while those of downregulated miRNAs were compared with upregulated DEGs using the Venny tool. There were five common DEGs and miRNA targets between downregulated miRNAs and upregulated DEGs, while there were four between upregulated miRNAs and downregulated DEGs (Fig 1, Table 2). The identified miRNAs were also verified using miRDIP online tool which uses over 30 miRNA target predicting tools to produce scores and ranking for the predicted miRNA targets. From the tool, six had scores of very high (top 1%), predicted by over ten tools and these are targets from hsa-miR-495(CDH2), hsa-miR-382(FGA), hsa-miR-20a(MMP2, MMP3 and CTSK) and hsa-miR-449a (P2RY14), two had high scores (top 5%), predicted by over 7 tools, and these are hsa-miR-619 (KNG1) and hsa-miR-576-5p (F9) and one scored medium (top 1/3) predicted by 6 sites, hsa-miR-619 (ALDH8A1) (Tables 3 and 4).

**Table 1:** Common differentially expressed miRNAs between GEO dataset GSE54088 and GSE56350.

*Common upregulated miRNAs*						
	**GSE54088**			**GSE56350**		
**miRNA**	**FDR^#^**	**P-value**	**FC^#^**	**FDR^#^**	**P-value**	**FC^#^**
hsa-miR-122	0.005	0.00004	4.025	0.00000002	0.00000000003	4.904
hsa-miR-20a	0.077	0.001	0.254	0.0139	0.0005	3.087
hsa-miR-107	0.039	0.0003	1.038	0.001	0.00002	2.524
hsa-miR-30b	0.048	0.0005	0.308	0.0008	0.000006	2.464
hsa-miR-30e	0.061	0.0007	0.848	0.014	0.0005	1.331
hsa-miR-27a	0.077	0.001	0.232	0.072	0.008	0.940
hsa-miR-425*	0.037	0.0002	1.478	0.026	0.002	0.689
hsa-miR-449a	0.095	0.002	0.607	0.029	0.002	0.448
hsa-miR-193a-3p	0.096	0.004	0.464	0.061	0.007	0.375
hsa-miR-17*	0.039	0.0003	0.942	0.081	0.010	0.315
hsa-miR-142-5p	0.037	0.0002	1.921	0.081	0.010	0.177
hsa-miR-19b	0.037	0.0001	1.701	0.082	0.010	0.148
hsa-miR-550*	0.081	0.006	0.453	0.010	0.012	0.134
hsa-miR-296-3p	0.081	0.006	0.191	0.010	0.012	0.111
*Common Downregulated miRNAs*						
hsa-miR-576-5p	0.040	0.002	-1.111	0.042	0.004	-0.744
hsa-miR-218	0.011	0.0002	-0.922	0.035	0.003	-0.182
hsa-miR-382	0.041	0.002	-0.711	0.096	0.012	-0.475
hsa-miR-487b	0.003	0.00003	-0.687	0.012	0.0004	-0.450
hsa-miR-645	0.003	0.003	-0.434	0.072	0.008	-0.577
hsa-miR-198	0.092	0.006	-0.324	0.035	0.003	-0.746
hsa-miR-608	0.060	0.003	-0.313	0.081	0.010	-0.276
hsa-miR-453	0.007	0.0001	-0.307	0.096	0.013	-0.080
hsa-miR-495	0.037	0.001	-0.248	0.096	0.012	-0.118
hsa-miR-619	0.099	0.007	-0.224	0.012	0.001	-0.900
hsa-miR-484	0.072	0.004	-0.210	0.081	0.010	-0.329
hsa-miR-574-3p	0.092	0.006	-0.204	0.081	0.010	-0.240
hsa-miR-612	0.092	0.006	-0.143	0.081	0.010	-0.333
hsa-miR-145	0.092	0.006	-0.127	0.012	0.0005	-0.739
hsa-miR-488*	0.099	0.007	-0.081	0.080	0.009	-5.021
hsa-miR-637	0.099	0.007	-0.071	0.096	0.012	-0.157

^#^FDR = False discovery rate, FC = log2 Fold Change

*indicates miRNAs that originates from the same hairpin structure of the main miRNA and is from the opposite arm of the precursor

**Table 2:** The common miRNAs and their common gene targets.

***Downregulated miRNAs***			
**miRNA**	**Precursor**	**Target**	**Gene Name**
miR-495	miR-495-3p	CDH2	cadherin 2, type 1, N-cadherin (neuronal)
miR-619	miR-619-3p	KNG1	kininogen 1
	miR-619-5p	ALDH8A1	aldehyde dehydrogenase 8 family, member A1
miR-382	miR-382-5p	FGA	fibrinogen alpha chain
miR-576-5p	miR-576-5p	F9	coagulation factor IX
***Upregulated miRNAs***			
miR-20a	miR-20a-5p	CTSK	cathepsin K
		MMP2	matrix metallopeptidase 2 (gelatinase A, 72kDa gelatinase, 72kDa type IV collagenase)
		MMP3	matrix metallopeptidase 3 (stromelysin 1, progelatinase)
miR-449a	miR-449a	P2RY14	purinergic receptor P2Y, G-protein coupled, 14

**Table 3:** The verification of the upregulated miRNAs and their targets in miRDIP tool.

miRNA	Precursor	Targets	Score	Class	Source No.	Sources
hsa-miR-20a	miR-20a-5p	MMP2	0.70	Very high	15	BiTargeting, DIANA, ElMMo3, MBStar, MAMI, microrna.org, MirAncesTar, miRDB, MirMAP, miRTar2GO, Mirza-G, MultiMiTar, RepTar, TargetRank, TargetScan
		CTSK	0.55	Very high	10	Cupid, DIANA, ElMMo3, MBStar, microrna.org, MirAncesTar, miRDB, MirMAP, Mirza-G, TargetScan
		MMP3	0.47	Very high	13	CoMeTa, DIANA, ElMMo3, MBStar, microrna.org, MirAncesTar, MirMAP, miRTar2GO, Mirza-G, MultiMiTar, RepTar, RNA22, RNAhybrid
hsa-miR-449a	miR-449a	P2RY14	0.39	Very high	12	BCmicrO, DIANA, ElMMo3, MBStar, microrna.org, MirAncesTar, miRDB, MirMAP, Mirza-G, RepTar, RNAhybrid, TargetRank

**Table 4:** The verification of the downregulated miRNAs and their targets in miRDIP tool.

miRNA	Precursor	Targets	Score	Class	Source No.	Sources
hsa-miR-495	miR-495-3p	CDH2	0.64	Very high	15	BiTargeting, DIANA, ElMMo3, MBStar, MAMI, microrna.org, MirAncesTar, miRDB, MirMAP, miRTar2GO, Mirza-G, MultiMiTar, RepTar, TargetRank, TargetScan
hsa-miR-382	miR-382-5p	FGA	0.49	Very high	12	BCmicrO, DIANA, ElMMo3, MBStar, microrna.org, MirAncesTar, miRDB, MirMAP, MirTar, Mirza-G, RNAhybrid, TargetRank
hsa-miR-619	miR-619-3p	KNG1	0.19	High	7	BCmicrO, CoMeTa, ElMMo3, MirAncesTar, miRbase, miRDB, RNAhybrid
	miR-619-5p	ALDH8A1	0.10	Medium	6	BCmicrO, DIANA, MBStar, MirAncesTar, MirMAP, RNAhybrid
hsa-miR-576-5p	miR-576-5p	F9	0.34	High	10	BCmicrO, CoMeTa, DIANA, ElMMo3, MBStar, MirAncesTar, miRDB, MirMAP, Mirza-G, TargetRank

### Clinical validation of the miRNAs

**Fig 2:**
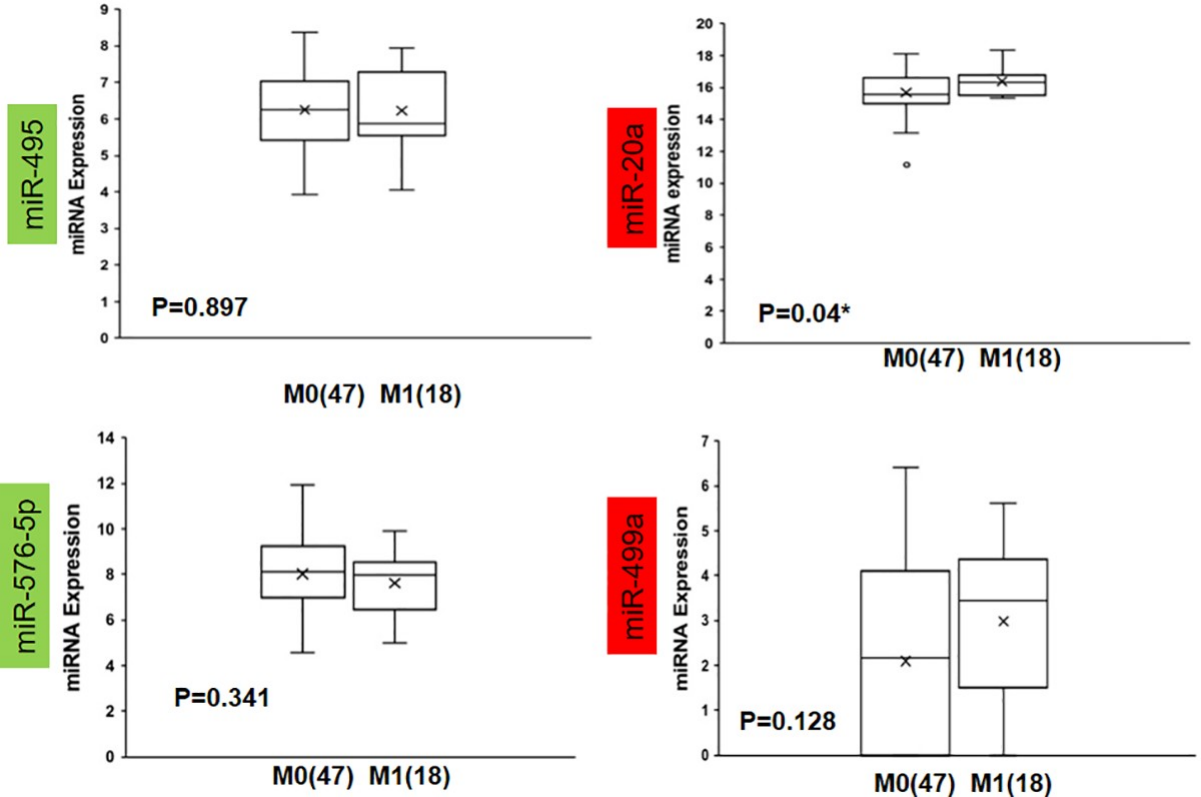
Expression patterns in GSE29623 of common miRNAs with targets from the DEGs. The miRNAs with a green background are commonly downregulated from Fig 1, and those with a red background are upregulated. The * represents Mann-Whitney U p-value<0.05, M0 = TNM stage I, II, III or no distant metastases and M1 = TNM stage IV or distance metastases; the number in brackets represents the case number. **Note:** miRNA-619 and miRNA-382 were not expressed in this dataset.

**Fig 3:**
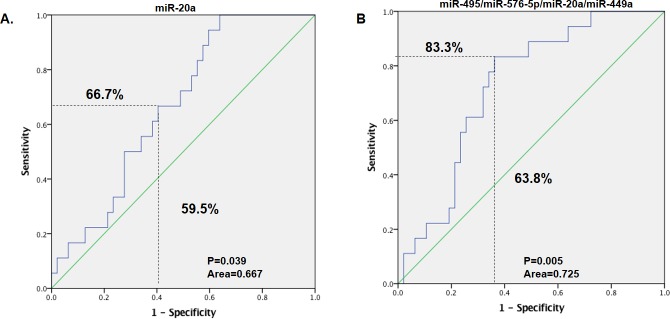
**The receiver operating characteristic (ROC) curve showing the area under the ROC curve (AUC), sensitivity and specificity of (A) miR-20a and (B) miRNA-495, miRNA-576-5p, miRNA-20a and miRNA-449a.** The regression model was estimated based on the miRNA of interest or the combination of miRNAs of interest, and the score was calculated for colorectal cancer (CRC) metastasis. The patients were divided into M0 and M1.

The common DE miRNAs which shared their targets with the identified common DEGS were validated in their roles to discriminate CRC patients with no distance metastases (M0) and those with distance metastases (M1). Therefore, the GSE29623 dataset was used to validate the common DE miRNAs in 65 cases of CRC, of which 47 were in stage M0 and 18 were in M1. Of the six common DE miRNAs, miR-619, which targets are ALDH8A1 and KNG1, and miR-382, which target is FGA, were not expressed. Among the expressed miRNAs, only miR-20a which targets are CTSK, MMP2 and MMP3 was significantly highly expressed in the patients with distant metastases (M1) as compared to those with no distance metastases (M0)(p = 0.04) while there was no significant difference in expression between M0 and M1 in the other three miRNAs (miR-495 its target is CDH2, miR-576-5p its target is F9, and miR-449a its target is P2RY14)) (Fig 2). Prediction performance was assessed by ROC analysis and summarized by the AUC. As a single classifier, hsa-miR-20a was the most promising, with a sensitivity of 66.7%, specificity of 59.5%, AUC of 0.667 and p-value of 0.039 (Fig 3A, Table 5). To further evaluate if the combination of the miRNAs can increase the sensitivity and specificity in predicting distance metastases, a logistic regression was run and the significance was tested. The regression model was estimated based on the two or more miRNAs and the probability score was calculated representing distance metastasis (M1). This model was followed by receiver operating characteristic (ROC) analysis [36]. Area under the ROC curve (AUC) was calculated together with the threshold of score, by Youden’s method. It was found that of all the combinations of miRNAs tested, it was the combination of all the four present miRNAs (miR-20a, miR-495, miR-576-5p and miR-449a) in the dataset that improved the prediction value, with a sensitivity of 83.3%, specificity of 63.8%, AUC of 0.725 and p-value of 0.005 (Fig 3B, Table 5).

**Table 5:** Area under curves for miRNAs in non-metastatic (M0) and distance metastases (M1) tissues.

miRNA	AUC^#^	P-Value	CI^#^
miR-495	0.499	0.988	0.337–0.660
miR-576-5p	0.426	0.356	0.273–0.578
miR-20a	0.667	0.039	0.534–0.799
miR-449a	0.618	0.145	0.476–0.759
miR-20a and miR-495	0.680	0.026	0.546–0.814
miR-20a and miR-576-5p	0.709	0.009	0.581–0.837
miR-20a and miR-449a	0.690	0.018	0.565–0.816
miR-20a, miR-495and miR-576-5p	0.712	0.009	0.584–0.839
miR-20a, miR-495and miR-449a	0.689	0.019	0.558–0.821
miR-20a, miR-576-5p and miR-449a	0.725	0.005	0.600–0.849
miR-20a, miR-495, miR-576-5p and miR-449a	0.725	0.005	0.599–0.850

^#^AUC = Area under curve, CI = confidence interval

### Validation of the miRNAs in patients’ exosomes

**Fig 4:**
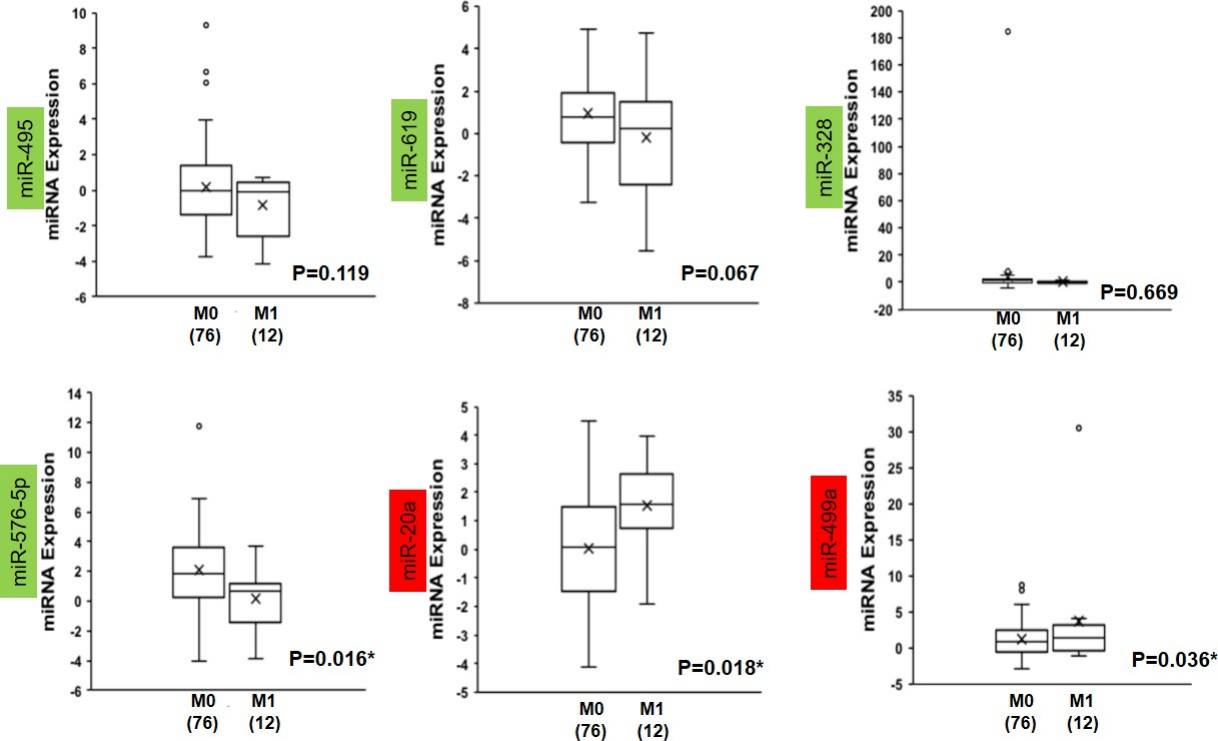
Expression patterns in the GSE39833 miRNA exosome dataset of common miRNAs with targets from the DEGs. The miRNAs with a green background are commonly downregulated from Fig 1, and those with a red background are upregulated. The * represents a Mann-Whitney U p-value<0.05, M0 = TNM stage I, II, III or no distant metastases and M1 = TNM stage IV or distance metastases; the number in brackets represents the case number.

Exosomes, small membrane vesicles secreted from various cells with their components, including miRNAs, reflect the original cells secreting them[44–47]; therefore, identifying exosomal miRNAs that can predict CRC progression to liver metastases can be very useful for clinical application. The GEO dataset GSE39833, comprising miRNA profiles from exosomes of 88 patients with CRC, of which 76 had no distant metastasis (M0) and 12 had distant metastasis (M1), was used to verify the common miRNAs. Among the downregulated miRNAs, miR-576-5p, whose target is F9, was significantly expressed at low levels (p = 0.016) in the M1 group compared with the M0 group, while there was no significant difference in the expression of miR-495, miR-619 and miR-382, which regulate CDH2, KNG1 and ALDH8A1, and FGA in the two groups (Fig 4). For the upregulated miRNAs, both miR-20a (CTSK, MMP2 and MMP3) and miR-449a (P2RY14) were significantly upregulated (p = 0.018 and 0.036, respectively) in the M1 group compared to the M0 group (Fig 4).

**Fig 5:**
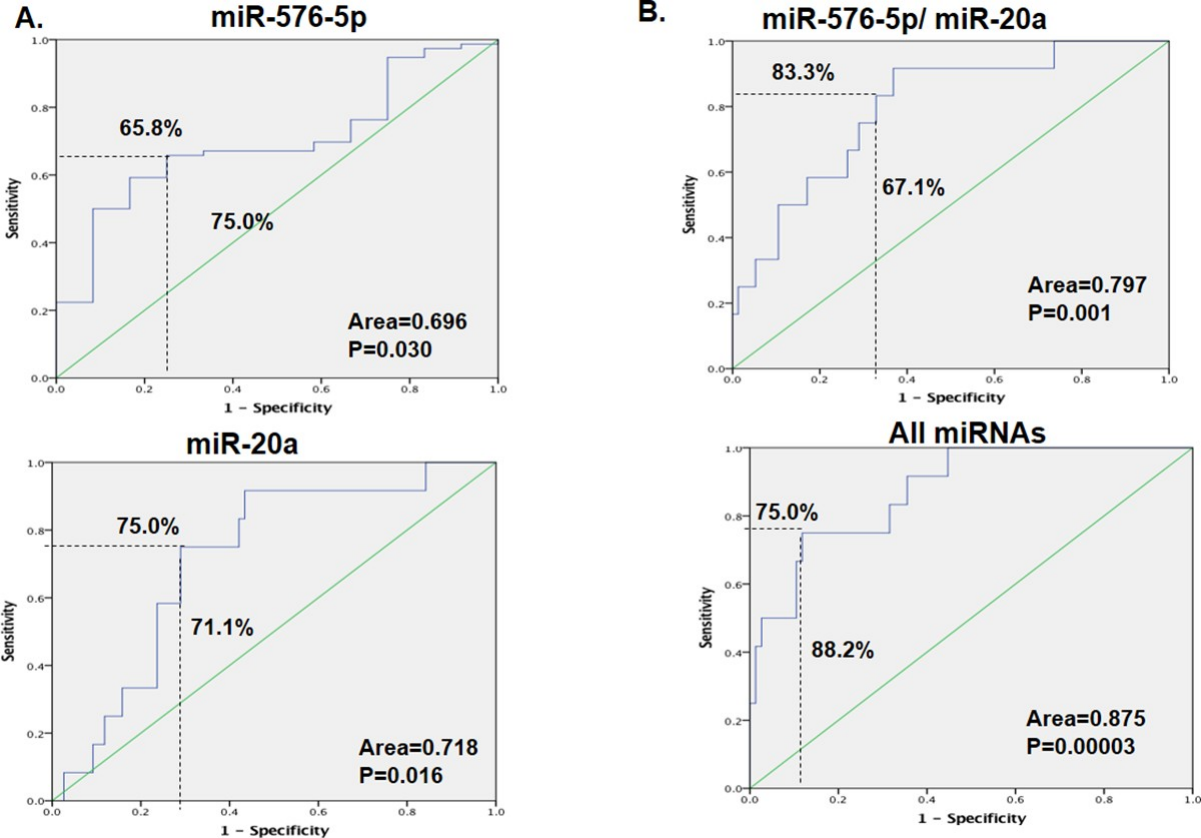
**The receiver operating characteristic (ROC) curve showing the area under the ROC curve (AUC), sensitivity and specificity of (A) miR-576-5p and miR-20a, (B) the combination of miR-576-5p and miR-20a and the combination of all six miRNAs.** The regression model was estimated based on the miRNA of interest or the combination of miRNAs of interest, and the score was calculated for colorectal cancer (CRC) metastasis. The patients were divided into M0 and M1.

The prediction performance by ROC analysis showed that as a single classifier, miR-576-5p and miR-20a had significant predictive values with sensitivities of 65.8% and 75.0%, specificities of 75.0% and 71.1%, AUCs of 0.696 and 0.718, and p-values of 0.030 and 0.016, respectively (Fig 5A, Table 4). The combination of the two miRNAs improved the predictive power, with a sensitivity of 83.3%, specificity of 67.1%, AUC of 0.797 and p-value of 0.001 (Fig 5B, Table 6). When all six miRNAs were combined, the prediction value increased further, with a sensitivity of 75.0%, specificity of 88.2%, AUC of 0.875 and p-value of 0.00003 (Fig 5B, Table 6).

**Table 6:** Area under curves for miRNAs in non-metastatic (M0) and distance metastases (M1) exosomes.

miRNA	AUC^#^	P-value	CI^#^
miR-495	0.609	0.229	0.455–0.762
miR-619	0.604	0.248	0.421–0.787
miR-382	0.559	0.511	0.405–0.714
miR-576-5p	0.696	0.030	0.559–0.834
miR-20a	0.718	0.016	0.581–0.855
miR-449a	0.567	0.458	0.386–0.747
miR-576-5p and miR-495	0.748	0.006	0.612–0.884
miR-576-5p and miR-619	0.785	0.002	0.679–0.891
miR-576-5p and miR-382	0.705	0.023	0.568–0.842
miR-576-5p and miR-449a	0.709	0.020	0.560–0.859
miR-576-5p and miR-20a	0.797	0.001	0.669–0.925
miR-20a and miR-495	0.731	0.010	0.595–0.867
miR-20a and miR-619	0.769	0.003	0.631–0.906
miR-20a and miR-382	0.734	0.010	0.600–0.867
miR-20a and miR-449a	0.741	0.007	0.598–0.885
miR-576-5p, miR-20a and miR-495	0.804	0.001	0.673–0.934
miR-576-5p, miR-20a and miR-619	0.845	0.0001	0.746–0.945
miR-576-5p, miR-20a and miR-382	0.805	0.001	0.677–0.932
miR-576-5p, miR-20a and miR-449a	0.797	0.001	0.665–0.930
miR-576-5p, miR-20a, miR-495 and miR-619	0.860	0.00007	0.759–0.960
miR-576-5p, miR-20a, miR-495 and miR-382	0.799	0.001	0.667–0.932
miR-576-5p, miR-20a, miR-495 and miR-449a	0.810	0.001	0.676–0.945
miR-576-5p, miR-20a, miR-619 and miR-382	0.844	0.0001	0.740–0.949
miR-576-5p, miR-20a, miR-619 and miR-449a	0.848	0.0001	0.748–0.948
miR-576-5p, miR-20a, miR-382 and miR-449a	0.795	0.001	0.659–0.931
miR-576-5p, miR-20a, miR-495, miR-619 and miR-382	0.865	0.00005	0.768–0.962
miR-576-5p, miR-20a, miR-495, miR-619 and miR-449a	0.867	0.00005	0.767–0.968
miR-576-5p, miR-20a, miR-495, miR-619, miR-382 and miR-449a	0.875	0.00003	0.779–0.971

^#^AUC = Area under curve, CI = confidence interval

### Clinical validation of the common miRNA targets

**Fig 6:**
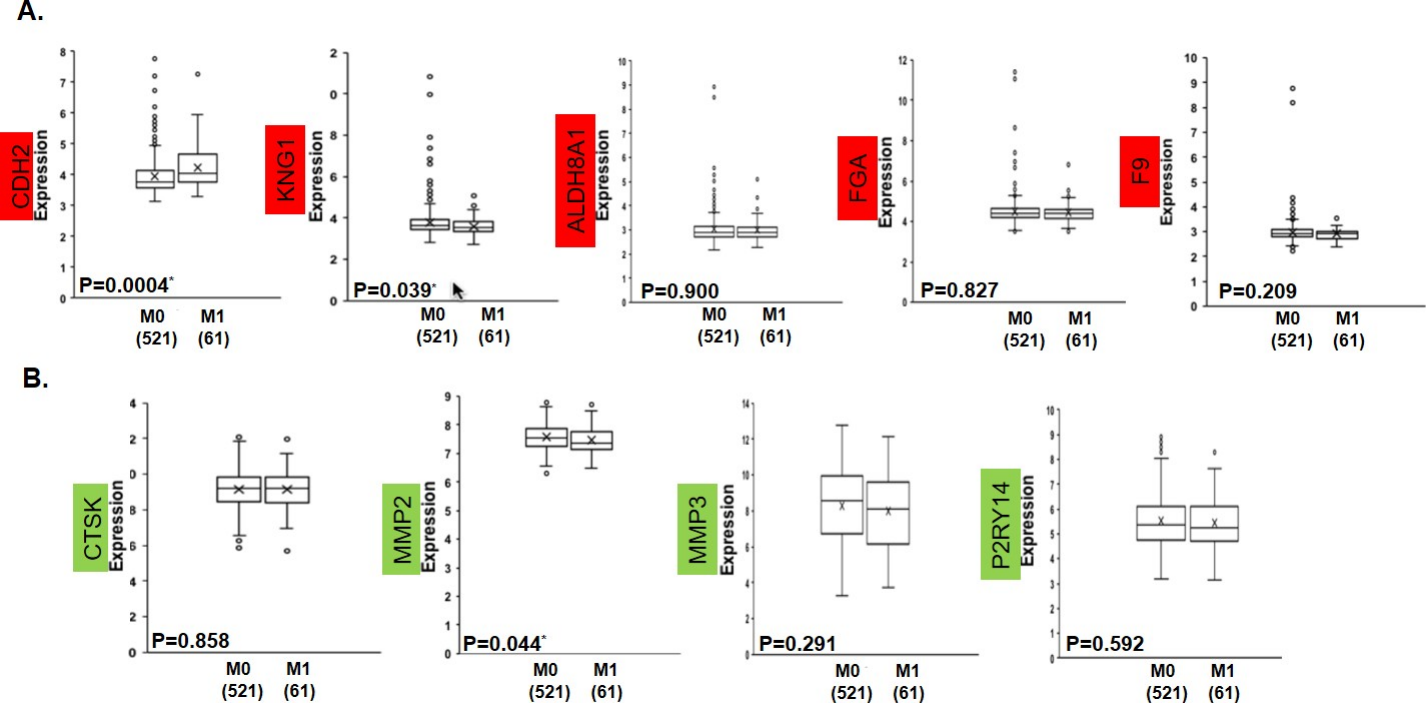
Expression patterns of the common miRNA targets in GSE39582. The miRNA targets with a red background are the commonly upregulated DEGs from Fig 1, and those with a green background are downregulated. The * represents a Mann-Whitney U p-value<0.05, M0 = TNM stage I, II, III or no distant metastases and M1 = TNM stage IV or distance metastases; the number in brackets represents the case number.

**Fig 7:**
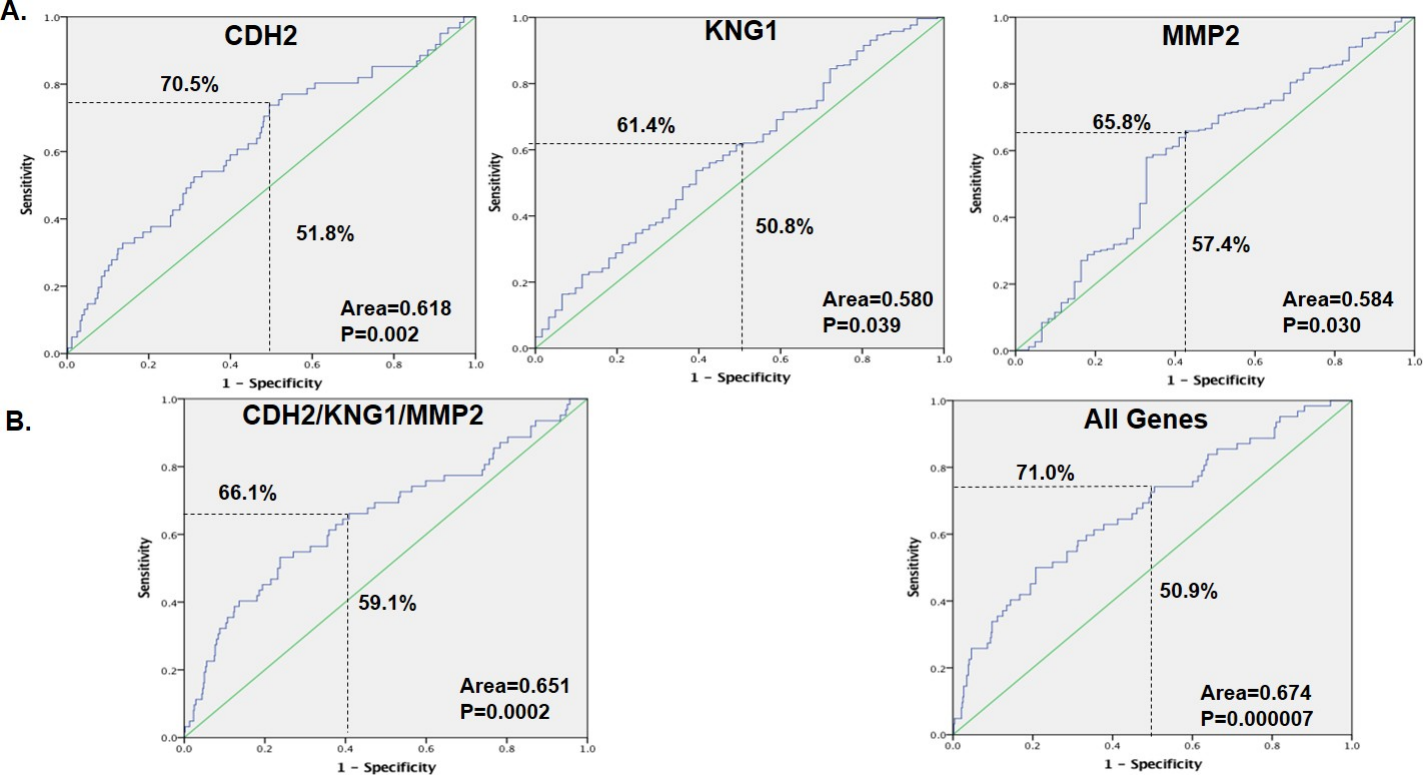
**The receiver operating characteristic (ROC) curve showing the area under the ROC curve (AUC), sensitivity and specificity of the miRNA targets (A) CDH2, KNG1 and MMP2, (B) a combination of CDH2, KNG1 and MMP2, and a combination of all nine miRNA targets.** The regression model was estimated based on the gene of interest or the combination of the genes of interest, and the score was calculated for colorectal cancer (CRC) metastasis. The patients were divided into M0 and M1.

The GSE39582 dataset comprising mRNA expression patterns from 598 tissues from patients with CRC, of which 521 had no distant metastasis (M0) and 61 (M1) had distant metastasis, was used to verify the common miRNA targets. For the targets whose miRNAs were downregulated, only CDH2 was significantly highly expressed in M1 (p = 0.0004), while KNG1 showed significant low expression (p = 0.039), but there were no significant differences in ALDH8A1, FGA and F9 (Fig 6A). For the targets for upregulated miRNAs, only MMP2 (p = 0.044) was significantly lowly expressed in M1, while there were no significant changes in CTSK, MMP3 and P2RY14 (Fig 6B). In the assessment of the prediction performance by ROC, CDH2 (sensitivity of 70.5%, specificity of 51.8%, AUC of 0.618 and p-value of 0.002), KNG1 (sensitivity of 61.4%, specificity of 50.8%, AUC of 0.580 and p-value of 0.039) and MMP2 (sensitivity of 65.8%, specificity of 57.4%, AUC of 0.584 and p-value of 0.030) had significant predictive values as single classifiers (Fig 7A). When the three genes were combined, the predictive value increased slightly (sensitivity of 66.1%, specificity of 59.1%, AUC of 0.651 and p-value of 0.0002), but the combination of all the target genes increased the predictive power more (sensitivity of 71.0%, specificity of 50.9%, AUC of 0.674 and p-value of 0.000007) (Fig 7B).

### Functional interaction and pathway enrichment

**Fig 8:**
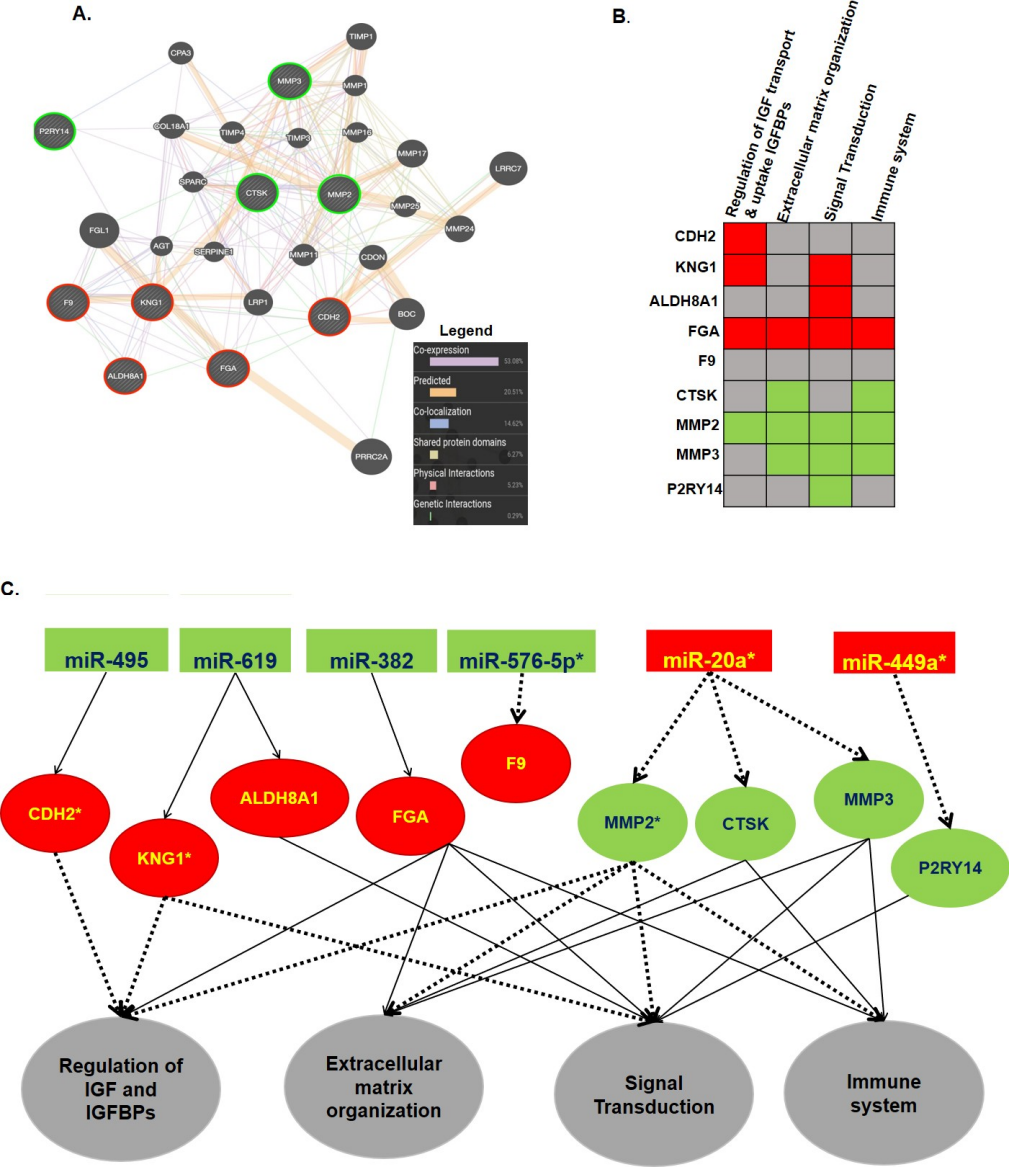
**Gene interaction network (A) and pathway enrichment summary of the common miRNA targets.** For Figure (A), the input genes are indicated with stripes, and the green circles represent downregulated and red represents upregulated genes in CRC liver metastases, while for (B), green indicates downregulation, red indicates upregulation and gray indicates that the gene was not enriched in that pathway. The pathways are in order of their enrichment value from left to right (genes≥4, FDR<0.05, p-value<0.05). (C) Schematic summary showing possible interactions of the miRNAs and their targets in their respective pathways. Red background represents upregulation and green background indicates downregulation in CRC liver metastasis, * represents significant expression in the respective validation dataset, thick dashed black lines represent significant interactions and thin black lines represent a nonsignificant interaction. The respective pathways are represented by a gray background.

The GeneMANIA online webtool was used to explore the functional interactions of the miRNA targets with each other, and the reactome was used to analyze the functional roles of these molecules. The interaction network included 20 other related genes in addition to the nine targets that were entered, and there were a total of 317 links. Six interaction types were involved, with coexpression being the most frequent type of interaction (53.08%) (Fig 8A). The target genes were also entered into the reactome Cytoscape software, and the reactome Fiz plugin was used to analyze the pathways. The pathways that contained ≥4 target genes, with an adjusted p-value <0.05 and p-value <0.05, were selected, and four pathways; regulation of Insulin-like Growth Factor (IGF) transport and uptake by Insulin-like Growth Factor Binding Proteins (IGFBPs), extracellular matrix organization, signal transduction and immune system, were enriched. Only F9 was not involved in the pathways (Fig 8B, Table 7). The whole interaction from the miRNAs to the target genes and their pathways is summarized in Fig 8C.

**Table 7:** Enriched Reactome pathways.

Pathway	P-value	FDR^#^	Genes
Regulation of Insulin-like Growth Factor (IGF) transport and uptake by Insulin-like Growth Factor Binding Proteins (IGFBPs)(R-HSA-381426)	2.20E-06	1.12E-04	CDH2, KNG1, FGA, MMP2
Extracellular matrix organization(R-HSA-1474244)	9.14E-05	0.001	FGA, CTSK, MMP2, MMP3
Signal Transduction (R-HSA-162582)	0.0009	0.016	KNG1, ALDH8A1, FGA, MMP2, MMP3, P2RY14
Immune system(R-HSA-168256)	0.009	0.031	FGA, CTSK, MMP2, MMP3

^#^FDR = False discovery rate

## Discussion

CRC metastases are a major obstacle in the clinical management of CRC. Liver metastases can be treated if detected early. Thus, this study used an integrated bioinformatic approach by applying patient tissues and samples to not only establish a model that can predict primary to liver metastases but also determine the pathways involved. The interactions of miRNAs result in changes in the transcription of their target genes[30]. Further analysis and the use of patient exosomes showed that this model can be applied in clinical settings, as these molecules are conserved and reflect the cells from which they were secreted. Exosomes are extracellular vesicles that play a role in the transport of bioactive molecules, including miRNAs[52]. They are secreted from all types of cells and are present in all biological fluids[52]. As exosomes participate in intercellular communication, their miRNAs have shown physiological and pathological functions in processes such as immune regulation and cancer progression[53–55].

In this study, although miR-576-5p and miR-20a showed promise in identifying patients who can develop distant metastases as single classifiers, the combination of the two and above all six miRNAs offered a far superior model (Fig 5). Wang et al. found that miR-576-5p is highly expressed in the serum of patients with CRC compared to that of the healthy controls, and they also observed that it is inversely correlated with cancer differentiation. miR-576-5p was highly expressed in TNM stage III and TNM IV cancer compared to that in stages I/II, and in lymphatic metastatic colon cancer[56], it has also been associated with CRC brain metastasis[57]. However, our study is the first to our knowledge to identify the association with liver metastases. Furthermore, we established that miR-576-5p may influence CRC progression to liver metastasis by targeting the F9 gene. Although the roles of F9 have been extensively explored in hemophilia[58, 59], there are very few studies that have investigated its roles in cancer progression. This lack of research partly explains the failure of the online pathway predictors to classify F9 into a specific pathway together with the other miRNA targets found in this study (Fig 8B and 8C); therefore, this pathway interaction requires further study.

MiR-20a has also been associated with CRC diagnosis, with high expression in stage IV cancer, and was predicted to respond to different chemotherapeutic agents. MiR-20a promotes proliferation, invasion and the epithelial-to-mesenchymal transition (EMT) of CRC cell lines. Similar to the results in this study, miR-20a was found to downregulate matrix metalloproteinases, including MMP2 and the EMT marker E-cadherin[60–63], supporting the findings of this study, although no study has linked it with the colonization of the primary tumor to the liver. Likewise, although miR-449a deficiency was shown to promote colon carcinogenesis[64], it has not been previously linked to liver metastases. Therefore, this study provides new information on the roles of these miRNAs in CRC progression. Other miRNAs, such as miR-495, are known to influence CRC cell proliferation, invasion, migration and EMT[65, 66]. MiR-495 was also found to directly affect the expression of invasion-related molecules, including MMP2[65]. As the tumor metastatic potential is a result of the balance between MMPs and TIMP-2, which plays a role in invasion by stimulating the degradation of the extracellular matrix (ECM) in CRC cells, these findings are logical, as we also established that CDH2 or N cadherin, an EMT marker, was directly regulated by miR-495. MiR-619 has been linked with prostate cancer diagnosis and the dissemination of the cancer beyond the capsule[67, 68], but no studies have linked it with CRC metastases before. MiR-382 inhibits cell growth, migration, invasion and enhanced chemosensitivity in CRC[69, 70].

We also identified predicted miRNA targets that were not reported in other studies; our analyses were performed with patient tissues and were able to replicate the expression of their regulating miRNAs. Although some targets were already known to play a role in metastasis of CRC, we further established their regulatory miRNAs and the pathways involved. Then, the best model to predict metastases was established, although it was not superior to that of the miRNAs (Fig 7). The explanation for the inconsistencies between miRNAs and their gene targets may be that the validation datasets did not specifically include those who had liver metastases but comprised all patients in stage IV, which includes all cases with distant metastases. This has been the case because of scarcity of miRNA and gene expression profiles which contain specific patient information on who developed liver metastases and from those who don’t in the follow up. But still this model is valid as over 70% of distance metastases from CRC are to the liver[40, 71], and therefore can be applied to predict patients with non-metastatic disease who may develop liver metastases in the long run. The other reason is that exosome expression cannot replicate tissue expression, and we cannot ignore the heterogenicity of CRC. However, despite these limitations, this study offers a mechanism for CRC progression to liver metastases. Our study demonstrated that during metastasis, there is an interaction of different pathways that involve different genes. A previous study established that CRC progression to liver colonization involves extracellular matrix organization[72, 73], and another study established that IGF regulation is also involved[74, 75]. We showed that the interaction of extracellular matrix organization, regulation of IGFs and IGBPs, signal transduction and the immune system pathways result in CRC metastasis to the liver. Lastly, as this study integrated different microarray datasets, the issue of systematic and technical bias arises as a result of different handling procedures. To address this issue, first only datasets generated using the same chip were considered for inclusion[76]. Therefore, all the gene datasets were generated using affrimetrix chips, with GSE49355 and GSE41258 generated on [HG-U133A] Affymetrix Human Genome U133A Array[16, 33], and GSE81558 on [PrimeView] Affymetrix Human Gene Expression Array[34]. However, due to the limited numbers of available miRNA datasets, all datasets which met the criteria for the study were included regardless of platform, and selected differentially expressed miRNAs and genes to try to minimize technical bias[77]. With this approach, the findings of this study provide clear insight into the mechanisms underlying CRC progression to liver metastases.

## Supporting information

S1 Table Upregulated differentially expressed genes (DEGs) in GSE49355, GSE41258 and GSE81558. (XLSX)

S2 Table Downregulated differentially expressed genes (DEGs) in GSE49355, GSE41258 and GSE81558. (XLSX)

S3 Table Common upregulated differentially expressed genes (DEGs) between GSE49355, GSE41258 and GSE81558. (XLSX)

S4 Table Common downregulated differentially expressed genes (DEGs) between GSE49355, GSE41258 and GSE81558. (XLSX)
